# Variant G57E of Mannose Binding Lectin Associated with Protection against Tuberculosis Caused by *Mycobacterium africanum* but not by *M. tuberculosis*

**DOI:** 10.1371/journal.pone.0020908

**Published:** 2011-06-10

**Authors:** Thorsten Thye, Stefan Niemann, Kerstin Walter, Susanne Homolka, Christopher D. Intemann, Margaret Amanua Chinbuah, Anthony Enimil, John Gyapong, Ivy Osei, Ellis Owusu-Dabo, Sabine Rüsch-Gerdes, Rolf D. Horstmann, Stefan Ehlers, Christian G. Meyer

**Affiliations:** 1 Department of Molecular Medicine, Bernhard Nocht Institute for Tropical Medicine, Hamburg, Germany; 2 Institute of Medical Biometry and Statistics, University Hospital Schleswig-Holstein, Campus Lübeck, Lübeck, Germany; 3 National Reference Center for Mycobacteria, Research Center Borstel, Borstel, Germany; 4 Microbial Inflammation Research, Research Center Borstel, Borstel, Germany; 5 Health Research Unit, Ghana Health Service, Accra, Ghana; 6 Kumasi Centre for Collaborative Research in Tropical Medicine, Kumasi, Ghana; 7 Deptartment of Community Health, School of Medical Sciences, Kwame Nkrumah University of Science and Technology, Kumasi, Ghana; 8 Cluster of Excellence Inflammation at Interfaces, Institute for Experimental Medicine, Kiel, Germany; Queen Mary University of London, United Kingdom

## Abstract

Structural variants of the Mannose Binding Lectin (MBL) cause quantitative and qualitative functional deficiencies, which are associated with various patterns of susceptibility to infectious diseases and other disorders. We determined genetic MBL variants in 2010 Ghanaian patients with pulmonary tuberculosis (TB) and 2346 controls and characterized the mycobacterial isolates of the patients. Assuming a recessive mode of inheritance, we found a protective association between TB and the *MBL2* G57E variant (odds ratio 0.60, confidence interval 0.4–0.9, *P* 0.008) and the corresponding LYQC haplotype (*P*_corrected_ 0.007) which applied, however, only to TB caused by *M. africanum* but not to TB caused by *M. tuberculosis*. In vitro, *M. africanum* isolates bound recombinant human MBL more efficiently than did isolates of *M. tuberculosis*. We conclude that MBL binding may facilitate the uptake of *M. africanum* by macrophages, thereby promoting infection and that selection by TB may have favoured the spread of functional MBL deficiencies in regions endemic for *M. africanum*.

## Introduction

Mannose-binding lectin (MBL) is a plasma opsonin of the collectin family. Its monomer consists of a 228-amino-acid polypeptide chain. Through extended alpha-helical structures it forms homotrimers which commonly associate to higher oligomers, preferentially hexamers. MBL plays a complex role in the immune response, in particular at primary contact with microbial pathogens but also in the later course infections. In a calcium-dependent fashion, it binds to carbohydrate structures on the surfaces of a wide range of microorganisms [1] and promotes phagocytosis either directly through a yet undefined receptor or indirectly via activation of the lectin pathway of complement.

Human MBL is encoded by the *MBL2* gene on chromosome 10 (10q11.2–q21; OMIM 154545), which comprises four exons. Its transcription is influenced by common promoter polymorphisms at nucleotide positions −550 (rs11003125, allele H>L), −221 (rs7096206, allele Y>X) and +4 (rs7095891, allele P>Q) [2]. Among these, the X allele (−221G) has a pronounced down-regulating effect. Of particular interest is a small cluster of structural variants at codon positions 52, 54 and 57 located in exon 1. The wild-type is designated allele A, and polymorphisms R52C (rs5030737), G54D (rs1800450) and G57E (rs1800451) are addressed as alleles D, B and C, respectively [3]. By disrupting the alpha-helical structure of the monomer they impede oligomerization. Heterozygous and homozygous variants result in partial or virtually complete functional deficiencies, respectively, whereby alleles B and C have stronger effects than allele D [4].

*MBL2* variants are quite frequent, although unevenly distributed in human populations [3]. Allele B is extremely rare in West Africa but occur at frequencies of 0.14, 0.25 and 0.50 in Caucasians, Asians and indigenous South Americans, respectively. In contrast, allele C is rare among Caucasians but common in sub-Saharan Africa, where it reaches frequencies of 0.20 and higher. Allele D in general is rather uncommon and appears to be largely restricted to North Africans and Caucasians. The high allele frequencies in certain populations suggested that functional MBL deficiencies might confer some biological advantage [5].

The genetic *MBL2* variants have been found to be associated with differences in susceptibility to a variety of infectious diseases and autoimmune disorders [4], [6]. Studies on human TB yielded partly inconsistent data. No significant associations were found in The Gambia but homozygosity of the C allele (G57E) tended to be negatively associated with pulmonary disease [7]. Similarly, the heterozygous occurrence of the B allele was found negatively associated with tuberculous meningitis in South Africa [8], and the B allele was also less frequent in Afro-American pulmonary TB cases than in controls [9]. In another study, however, the frequency of the B allele was significantly higher in Afro-American TB patients than in controls [9]. In the same study, no differences between cases and controls were seen in individuals of Caucasian and Hispanic origin while a further study found MBL low-producing haplotypes more frequent in Caucasian TB patients than among controls [10]. In South India, a positive association of the B allele with disease was observed [11], and likewise, the C allele was found to be more frequent among HIV-positive TB patients than among HIV-negative controls in Malawi [12]. Finally, no association of *MBL2* variants with TB was found in a Han Chinese population [13].

Evidence has been presented indicating that genetic variants of the pathogen may also significantly contribute to both acquisition and clinical progression of TB [14]–[17]. Mycobacteria causing “typical” TB have been summed up in the *M. tuberculosis* complex (MTBC), and, to differentiate the various isolates, a variety of genetic methods have been established including the so-called spoligotyping and IS*6110* mycobacterial fingerprinting, in addition to the assessment of mycobacterial growth on selective media and the morphology of colonies as well as conventional biochemical assays.

We have studied the influence of *MBL2* variants on susceptibility and resistance to TB by comparing HIV-negative patients with smear- and/or culture-positive pulmonary TB to unaffected and unrelated control individuals in Ghana, West Africa. Genotyping of MTBC isolates allowed to stratify in the association study for the infecting MTBC strains and to search for a contribution of pathogen variability.

## Results

### Genetic Association

A total of 2010 cases and 2346 controls were genotyped for the three structural *MBL2* exon 1 polymorphisms R52C (allele D), G54D (allele B) and G57E (allele C) and three additional polymorphisms at position −550 (alleles H and L) and −221 (alleles Y and X) in the promoter and at position +4 (alleles P and Q) of the untranslated region of exon 1. Allele C was found at a frequency of 0.33 in the entire study group. The D allele was not detected at all, and allele B occurred at a frequency of 0.006 and, therefore, was not considered in the statistical analyses. All variants were in Hardy-Weinberg equilibrium both in the case and control groups (Table 1).

**Table 1:** *MBL2* variants genotyped.

*MBL2* variants	rs #	alleles	alternative nomenclature	localization	contig (chromosome 10)	contig position	chromosomal position (bp)	MAF (%)	HWE cases *P*	HWE controls *P*
−550	rs11003125	G/C	H/L	Promoter	NG_008196.1	g.4447C>G	54202020	6.9	0.24	0.40
−221	rs7096206	C/G	X/Y	Promoter	NG_008196.1	g.4776C>G	54201691	12.4	0.83	0.50
+4	rs7095891	A/G	Q/P	Exon 1	NG_008196.1	g.5000C>T	54201467	38.8	0.57	0.19
G54D	rs1800450	A/G	A/D	Exon 1	NM_000242.2	c.161A>G	54201241	0.6	-	-
G57E	rs1800451	G/A	A/C	Exon 1	NM_000242.2	c.170G>A	54201232	33.1	0.75	0.46

rs, gene variant reference id; bp, base pairs; MAF, minor allele frequency; HWE, Hardy Weinberg equilibrium.

Out of a total of 1567 mycobacterial isolates that were genotyped, 1029 (65.7%) were *M. tuberculosis* Euro-American (EUAM), and 56 (3.6%) belonged to the *M. tuberculosis* East-African-Indian (EAI), Beijing or Delhi lineages. *M. africanum* was identified in 472 (30.1%) isolates (*M. africanum* West African 1, n = 336; *M. africanum* West African 2, n = 136), and 10 (0.6%) were *M. bovis*. As the *M. africanum* and *M. bovis* lineages are closely related phylogenetically by exhibiting both the RD9 deletion, they were considered collectively in statistical analyses.

The frequencies of *MBL2* variants found are shown in Table 2. No significant differences were observed between cases and controls when including all cases. However, stratification for the major MTBC species of *M. tuberculosis* and *M. africanum/M. bovis*, revealed that, assuming a recessive mode of inheritance, variant G57E (allele C) occurred at a significantly lower frequency in patients infected with *M. africanum/M. bovis* than in controls (OR 0.60, 95% CI 0.4–0.9, nominal *P* value [*P*_nom_] = 0.008), whereas no evidence for an association was found for *M. tuberculosis* patients alone. This distribution of genotypes implies that occurrence of the *MBL2* C allele is compatible with relative protection from TB caused by *M. africanum/M. bovis*.

**Table 2:** *MBL2* genotype associations of all TB cases, cases infected with *M. tuberculosis* and cases infected with *M. africanum*, compared with controls.

		All TB cases	*M. africanum/M. bovis* cases	*M. tuberculosis* Cases	Controls	All cases vs. controls	*M. africanum/M. bovis* cases vs. *controls*	*M. tuberculosis* cases vs controls
*MBL2* variant	genotype	%	%	%	%	OR (CI) *P*	OR (CI) *P*_nom_	OR (CI) *P*_nom_
−550	CC	85.2	87.0	85.2	86.4	1	1	1
	CG	14.4	12.6	14.4	13.2	1.09 [0.9–1.3] 0.36	0.93 [0.7–1.3] 0.66	1.08 [0.9–1.3] 0.51
	GG	0.4	0.4	0.4	0.4	1.04 [0.4–2.9] 0.94	1.31 [0.3–6.2] 0.73	1.15 [0.3–3.9] 0.83
		n = 1843	n = 446	n = 972	n = 2174			
−221	GG	77.3	76.2	76.5	76.3	1	1	1
	CG	21.3	21.8	22.4	22.3	0.93 [0.8–1.1] 0.34	0.96 [0.8–1.2] 0.77	0.99 [0.8–1.2] 0.94
	CC	1.4	2.0	1.1	1.4	0.97 [0.6–1.7] 0.91	1.40 [0.7–3.0] 0.39	0.79 [0.4–1.6] 0.51
		n = 1859	n = 449	n = 982	n = 2180			
+4	AA	37.1	36.0	37.9	37.0	1	1	1
	AG	47.1	47.9	46.7	48.7	0.95 [0.8–1.1] 0.48	0.99 [0.8–1.2] 0.91	0.93 [0.8–1.1] 0.37
	GG	158	16.1	15.4	14.3	1.09 [0.9–1.3] 0.36	1.15 [0.8–1.5] 0.37	1.05 [0.8–1.3] 0.69
		n = 1953	n = 478	n = 1033	n = 2230			
G57E	GG	46.7	48.9	46.9	44.8	1	1	1
	AG	43.0	44.2	41.0	43.7	0.95 [0.8–1.1] 0.42	0.92 [0.7–1.1] 0.43	0.90 [0.8–1.1] 0.19
	AA	10.2	6.9	12.1	11.5	0.90 [0.7–1.1] 0.33	0.58 [0.4–0.9] 0.006	1.07 [0.8–1.4] 0.60
						recessive model	**0.60 [0.4–0.9] 0.008**	
		n = 1894	n = 477	n = 1040	n = 2236			

Odds ratios (OR) and nominal *P* values (*P*_nom_) adjusted for gender, age and ethnic groups by logistic regression.

CI, 95% confidence interval.

**Figure 1:**
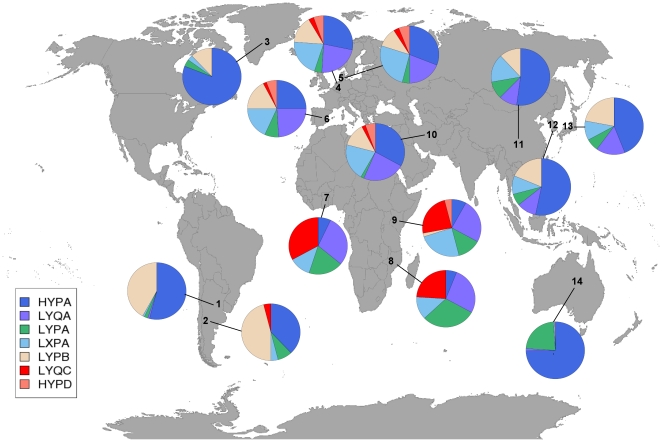
MBL2 haplotype frequencies in different populations. 1, Argentina (Chiriguanos) [2]; 2, Argentina (Mapuche) [2]; 3, Greenland [2]; 4, The Netherlands [47]; 5, Denmark [2]; 6, Spain [48], 7; Ghana (present study); 8, Mozambique [2]; 9, Kenya [2]; 10, Iran [49]; 11, China [13]; 12, Korea [50]; 13, Japan [51]; 14, Australia [52].

Haplotypes of promoter polymorphisms and the codon 57 variants (allele C) were inferred from unphased genotype data. The presumably ancient *MBL2* haplotype LYQC [5] (Table 3 and Fig. 1), comprising the most common exon 1 variant in Africa (allele C; our data and [18]), was strongly associated with protection from infections caused by lineages of the *M. africanum/M. bovis* species in a recessive model of inheritance (corrected *P* value [*P*_corr_] = 0.007 after 50 000 permutations; Max-Stats global *P* value 0.035).

**Table 3:** *MBL2* haplotype association comparing TB cases infected with *M. africanum* and controls.

Haplotype	MBL2 SNPs				Score statistic^1^	Freq. cases^2^	Freq. controls^2^	*P* 3	*P*_corr_ 4
	−550 G/C>H/L	−221 C/G>X/Y	+4 A/G>Q/P	G57E G/A>A/C					
1	L	Y	Q	A	−1.31	0.31	0.28	0.19	0.19
2	L	X	P	A	−1.12	0.13	0.12	0.26	0.24
3	H	Y	P	A	−0.53	0.07	0.07	0.60	0.52
4	L	Y	P	A	0.78	0.20	0.19	0.43	0.43
5	L	Y	Q	C	2.70	0.29	0.33	**0.007**	**0.007**

1 Score statistic of the haplo.score software.

2 Frequency; only haplotypes with a frequency >0.01 are shown.

3 *P* values of haplotype-specific associations with a recessive mode of inheritance.

4 Simulated haplotype-specific *P* values corrected for multiple testing by 50,000 permutations.

### Binding of MBL to MTBC Isolates

**Figure 2:**
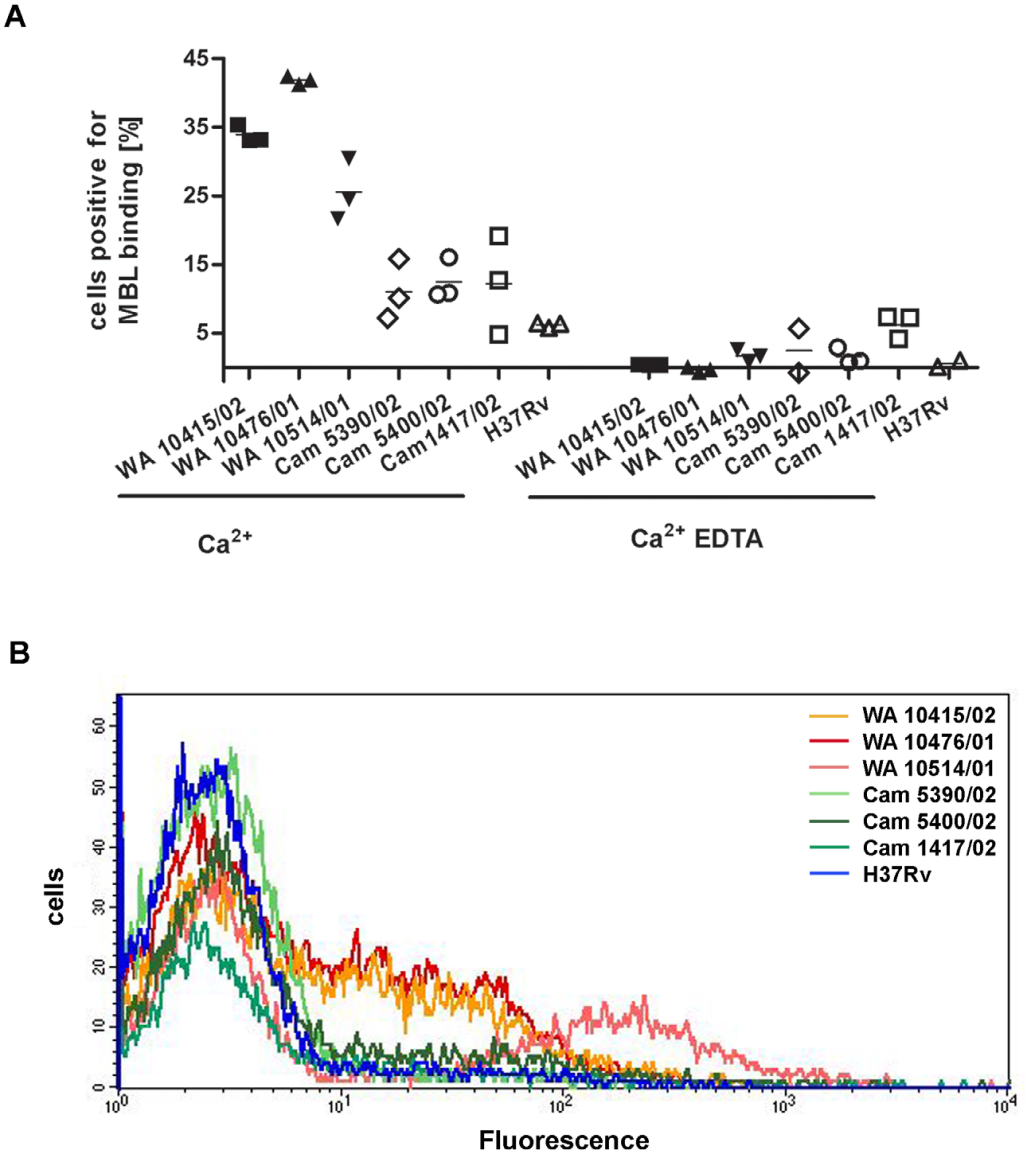
Comparison of MBL binding to *M. africanum* vs. *M. tuberculosis* strains by flow cytometry. Representative clinical isolates of *M. africanum* and *M. tuberculosis* strains were processed for flow-cytometric analysis by labeling with recombinant human MBL, biotinylated anti-human MBL antibody and Cy5-conjugated streptavidin). The percentage of positive cells for MBL binding was analyzed in triplicates for three different clinical isolates of *M. africanum* and *M. tuberculosis* along with the laboratory strain H37Rv. Calcium dependence of binding was examined by the addition of EDTA to the TBS/Ca^2+^ buffer. Negative controls were processed in the absence of MBL. The mean percentage of positive cells of these controls was subtracted from the corresponding percentages of positive cells of MBL-treated mycobacteria (A). Representative flow-cytometric profiles of MBL binding to clinical isolates (depicted in A) in the presence of Ca^2+^ (B). The Ghanaian MTBC strains used for the binding assays were *M. africanum* West African 2 strains 10415/02, 10476/01, 10514/01 and *M. tuberculosis* Cameroon strains 5390/02, 5400/02 and 1417/02.

Binding of MBL to glycosylated bacterial surfaces is a prerequisite for complement activation and initiation of phagocytosis. Flow cytometry was applied to study the interaction of MBL with *M. tuberculosis* and *M. africanum*. Mycobacterial suspensions were labelled with recombinant human MBL, biotinylated anti-MBL antibody and Cy5-coupled streptavidin, and the percentage of cells positive for MBL binding was assessed. For the three clinical isolates (different IS*6110* DNA fingerprints, data not shown) of *M. africanum* (West African 2 10415/02, 10476/01 and 10514/01), approximately 35% percent of the population bound MBL, whereas about 10% of the *M. tuberculosis* strains (Cameroon 5390/02, 5400/02, 1417/02) were positive for MBL binding. Only five percent of cells of the laboratory *M. tuberculosis* strain H37Rv were positive for MBL binding. In order to further evaluate whether binding was mediated by C-type lectin interactions, inhibition experiments using the calcium chelating agent EDTA were performed. Binding of MBL was clearly inhibited in the presence of 15 mM EDTA, demonstrating the divalent cation-dependent nature of MBL binding in this assay format (Fig. 2 A). Representative histograms of MBL binding to clinical isolates in the presence of Ca^2+^ demonstrate a significant increase in mean fluorescence intensity for the three *M. africanum* strains when compared to four *M. tuberculosis* strains (*P* = 0.034; Fig. 2 B).

In conclusion, all *M. africanum* strains tested bound MBL significantly better than did *M. tuberculosis* strains.

## Discussion

Quantitative and functional MBL deficiencies resulting from mutations in the promoter and the first exon of the *MBL2* gene are common throughout the human populations [18] (Fig. 1). We found in a recessive mode of inheritance that the *MBL2* low-producer haplotype LYQC including the structural variant G57E (allele C) was associated with protection from TB caused by *M. africanum/M. bovis* but not from TB caused by *M. tuberculosis*. In addition to low plasma MBL levels associated with the L, Y and Q promoter alleles, allele C is associated with lower amounts of the functionally important MBL oligomers [4].

The observation in the genetic analysis was corroborated by MBL binding studies that were performed with *M. tuberculosis* and *M. africanum* isolates originating from Ghanaian TB patients and with the laboratory strain H37Rv. The binding assays indicated that all *M. africanum* isolates tested bound recombinant human MBL to a greater extent than did *M. tuberculosis* isolates. Binding of MBL to mycobacteria may mediate phagocytosis by their target cell, the macrophage. Therefore, it may be concluded that a decreased concentration and a functional impairment of MBL caused by mutations of the LYQC haplotype might reduce phagocytosis of mycobacteria by macrophages. Accordingly, reduced uptake by macrophages could be one of a multitude of events that contribute to resistance against TB. This conclusion is supported by an experiment showing that a depletion of alveolar macrophages increased resistance to tuberculosis in a murine infection model indicating that uptake by macrophages serves the survival and propagation of the mycobacteria rather than their control by the host [19]. The observations made in this mouse model are, however, not directly valid in human disease.

As MBL bound less effectively to *M. tuberculosis in vitro* one may also hypothesize that impairment of MBL function is of less relevance in TB caused by lineages of this species. Since MBL is only one of several components which interact with mycobacterial surface moieties, other mediators and cellular receptors might be more important in the phagocytic uptake of M. tuberculosis [20]. In addition to MBL, the mannose receptor of macrophages (MRC1) as well as receptors for complement (CR1, CR3, CR4), surfactant protein A, macrophage scavenger receptors and CD14 all have been reported to be involved in pathogen-macrophage interactions [21]. Thus, alternative mechanisms of uptake of mycobacteria by macrophages exist and the responsiveness of macrophages does not exclusively depend on recognition of mycobacterial structures by MBL. It may, therefore, be concluded that some or all of these receptors are more relevant in *M. tuberculosis* than in *M. africanum/M. bovis* infections.

Genome analyses of several MTBC phylogenetic lineages have revealed a multitude of variations indicating that the members of the MTBC are far more genetically diverse than previously thought [22]. Genes of various functional categories such as those encoding constituents of metabolic pathways, membrane proteins and virulence factors show remarkable variability, and the various lineages are characterized by deep-tailed genomic deletions (regions of differences; RD) [15], [23]. So far, the effect of genetic variation on the composition of cell walls of *M. africanum/M. bovis* and *M. tuberculosis* is incompletely explored. In particular, it is not clear whether the RD9 deletion, which is characteristic for *M. africanum* lineages and for *M. bovis*, has an influence on the composition of cell surface structures which might be relevant to MBL binding. The binding characteristics of MBL to microbes are also not fully understood. It has, however, been shown that MBL variability affects its binding to mycobacteria [24], [25]. Interestingly, the TBd1 region, which is deleted in *M. tuberculosis* but not in *M. africanum/M. bovis* lineages, comprises a number of genes encoding membrane proteins which are thought to be responsible for lipid transport and might be involved in determining the composition of cell wall.

Differences in MBL binding properties have also been observed in other bacterial infections [1], [26]. Certain structures of capsular polysaccharides, for example, have been shown to decrease MBL binding [27] and bacterial cell wall components appear to be able to mask MBL ligands (e.g. mannose, N-acetylglucosamine) or to modify carbohydrate moieties or their densities on cell walls to prevent MBL binding [28]. Taken together, considerable variation exists in the strength of MBL binding to many microorganisms.

There is longstanding debate on whether MBL variability has evolved through neutral evolution or as a result of natural selection [29]–[31]. In a large study of more than 9000 Caucasians, MBL dependent effects on the morbidity or mortality from infectious diseases could not be identified [32]. Another study of febrile adults did not find any correlation between microbial infections and the MBL pathway of complement activation [33]. In contrast, a plethora of case-control association studies has attributed a role of MBL variability to infection phenotypes [34], and the common belief now is that *MBL2* variability has arisen as a consequence of human contact with pathogenic microorganisms in diverse geographic environments [18]. However, which balancing selection pressure, if any, might have established and preserved the high frequencies of MBL variants, in particular that of allele C in Africa, is not clear. It is also uncertain in how far mycobacterial infections may have contributed to disseminate the variant *MBL2* haplotypes and to shape human MBL diversity. It has repeatedly been suggested that mycobacterial infections might have contributed significantly to maintain MBL variability [35], [36]. The results of our candidate gene study are in line with these suggestions and argue for the emergence of MBL variability at least in part through selective forces. It is, however, unlikely that mycobacteria alone have exerted sustained selective pressure, as studies with infection phenotypes caused by other pathogens have also yielded significant genetic associations [4].

MBL variability appears to be of importance in infections with mycobacteria which can bind great amounts of MBL as documented in the present study. Apart from the mere genetic aspect of the question addressed in our study it is now also a matter of discussion whether deficiency of functional MBL may lead to relative protection through impaired uptake of mycobacteria or may cause increased susceptibility to TB [37]. There is, however, considerable debate on whether great or low amounts of MBL have any effect on the phenotype of MTBC infection and evidence on this issue is ambiguous [7]–[13]. Notably, in all previous association studies species and strain determination of MTBC agents and the corresponding statistical stratifications were not performed.

Recent studies suggest that the MTBC has evolved in East Africa approximately 40.000 years ago from a common ancestor, addressed as *M. prototuberculosis* [22], [38]. By human migration the ancient *M. africanum/M. bovis* lineages, which first evolved from its progenitor and are now characterized by the RD9 deletion, were directly disseminated across sub-Saharan Africa. The more modern *M. tuberculosis* lineages, characterized by the TBd1 deletion, moved out of Africa, spread initially over Europe and Asia, and were re-introduced to Africa only centuries ago where they are now part of the MTBC spectrum. This early geographical segregation is believed to be the cause of lineage-specific adaptation to distinct human host groups [22], [23].

The protective *MBL2* haplotype LYQC, which is virtually unique to sub-Saharan Africa and occurs there at high frequencies, might have been selected for, because it confers, with a significant odds ratio, protection from clinical TB caused by *M. africanum/M. bovis*, The question, however, namely that the *MBL2* C allele occurs across sub-Saharan Africa, while *M. africanum* appears still to be largely restricted to the West African region [39], remains to be solved. Indeed, *M. africanum* has been claimed to cause TB, although as an uncommon pathogen, in other parts of Africa as well. However, its occurrence outside of West Africa is not well documented. As MBL is a molecule involved in the immune response to a variety of pathogens, one explanation might be that associations exist with phenotypes of other infectious diseases.

Lastly, the preliminary evidence that MBL2 variability in Africa, in particular occurrence of the C allele, is associated with higher *Plasmodium falciparum* parasite counts and more severe courses of malaria [37], [34] raises the question of TB-malaria counterbalancing effects. Early selection of human genetic factors that provide protection against the ancient species *M. africanum* [22], [38] prior to the more recent emergence of *P. falciparum* [40] in Africa might now contribute to the deadly toll paid by falciparum malaria.

## Materials and Methods

### Ethics Statement, Patients and Controls

The study protocol was approved by the Committee on Human Research, Publications and Ethics of The School of Medical Sciences, Kwame Nkrumah University of Science and Technology, Kumasi, Ghana, and the Ethics Committee of the Ghana Health Service, Accra, Ghana. Blood samples were taken only after a detailed explanation of the aims of the study, and consent was obtained by signature or thumbprint.

Participants were consecutively enrolled in Ghana, West Africa, between September 2001 and July 2004 at Korle Bu Teaching Hospital in Accra, Komfo Anokye Teaching Hospital in Kumasi, plus 15 additional hospitals and polyclinics in Accra and Kumasi and at regional district hospitals. The case group included of 2010 HIV-negative individuals with smear-/culture-positive pulmonary TB. Out of a total of 2346 control individuals, 1211 were unrelated personal household contacts of cases and 1135 were individuals from neighbouring houses or working contacts of cases. Cases and controls belonged to the ethnic groups of Akan (Ashanti, Fante, Akuapem), Ga-Adangbe, Ewe, all in the south of Ghana, and several other ethnic groups from northern Ghana.

Patient phenotyping was based on the medical histories and the documentation of major TB symptoms on structured questionnaires, physical examination, HIV-1/2 antibody status testing (Capillus, Trinity Biotech, Bray, Co Wicklow, Ireland) and posterior-anterior chest X-rays. The microbiological diagnosis was made by Ziehl-Neelsen staining of two independent sputum smears and culturing of *M. tuberculosis* on Löwenstein-Jensen agar with subsequent determination of mycobacterial species, lineages and fine-typing of mycobacterial genotypes. Cases were HIV-negative and had radiological lesions characteristic of pulmonary TB. All patients were treated in the framework of the DOTS programme (Directly Observed Treatment Short-Course Strategy) organized by the National Tuberculosis Programme of Ghana. Study participants belonged to the following ethnic groups (cases/controls): Akan including Ashanti, Fante, Akuapem (63.6%/59.1%), Ga-Adangbe (14.5%/19.8%), Ewe (7.1%/9.3%) and ethnic groups of northern Ghana including Dagomba, Sissala, Gonja, and Kusasi (12.9%/10.4%). The proportions of ethnicities among patients and controls did not differ significantly.

Disclosure of HIV test results was dependent on the prior documentation of the willingness of the study participants to be informed and included for HIV-positive patients their prompt referral to counseling and treatment provided by the Ghanaian AIDS Control Programme.

The characterisation of controls consisted of a medical history and clinical examination, chest X-ray and a tuberculin skin test (Tuberculin Test PPD Mérieux, bioMérieux, Nürtingen, Germany). The controls had no radiological signs of actual or previous pulmonary TB. Further details of the recruitment procedure and the composition of the study group including the distribution of ethnicities have been described previously [41], [42].

### HIV Testing

For HIV-1/-2 testing of TB cases, a capillary test system (Capillus, Trinity Biotech, Bray, Co Wicklow, Ireland) was applied. HIV positivity was confirmed by the Organon Teknika Vironostika HIV-1/-2 EIA system (Organon Teknika, Turnhout, Belgium). The rate of confirmation was 100%. HIV-positive TB patients were excluded from further genetic analyses.

### Genotyping of Genetic Variants

After DNA extraction from peripheral blood by a magnetic separation technology (AGOWA® mag Maxi DNA Isolation Kit, Berlin, Germany) according to the manufacturer's instructions.

Genotyping of the three structural *MBL2* variants in exon 1 at codons 52, 54 and 57 (allele A = wild-type, alleles B, C, D = variant alleles) were performed using a high-throughput pyrosequencing assay (http://www.pyrosequencing.com/) at the Max-Delbrück-Center for Molecular Medicine, Berlin, Germany. Promoter variants at nt positions −550, −221 and +4 were analyzed by dynamic allele-specific hybridization with fluorescence resonance energy transfer (FRET) in a LightTyper device (Roche). Primer pairs and sensor/anchor oligonucleotides for LightTyper-based *MBL2* promoter genotyping are listed in Table 4.

**Table 4:** Primer Pairs and sensor/anchor oligonucleotides for LightTyper-based *MBL2* genotyping.

*MBL*2	Primer oligonucleotides	Sensor/Anchor oligonucleotides
−550	F-AGAAAGTAGAGAGGTATTTAGCAC	S-GGCAAGCCTGTCTAAAACACCAAG-6-Fam
	R-GTGATCCAAAGAGGAAGGTC	A-Cy5-GGAAGCAAACTCCAGTTAATTCTGGGCTGGG-phosphate
−221	F-CTCATTCTCATTCCCTAAGCTAAC	S-GCCACCGAAAGCATGTTTATAGTCT-6-Fam
	R-AACAAAGGTAGGCACTATGAT	A-Cy5-CCAGCAGCAACGCCAGGTGTC-phosphate
+4	F-GAGAAGGAGAGGGAGTGAT	S-Cy5-GCTCGGTAAATATGTGTTCATTAACTGAGATTAAC-phosphate
	R-GTGTCACAAGGAATGTTTACTTT	A-CTGCACCCAGATTGTAGGACAGAGGGC-6-Fam
p.G54D	F-GTGGCAGCGTCTTACTC	S-TGGGCGTGATGACACCAAGGAAGA-6-Fam
p.G57E	R-TTCCTCTGGAAGGTAAAGAATTG	A-Cy5-AAGGGGGAACCAGGTACGTGTTGGG-phosphate

F, forward primer; R, reverse primer; S, sensor oligonucleotide; A, anchor oligonucleotide; 6–Fam, 6–carboxyfluorescein; Cy5, corresponding to the Roche fluorophore LC Red 640; synthesized by biomers.net GmbH, Ulm, Germany.

### Typing of MTBC Agents

MTBC isolates were cultured on solid Löwenstein-Jensen media and shipped to the German National Reference Centre for Mycobacteria (Borstel, Germany) for minute analyses of biochemical, growth and molecular characteristics. Molecular differentiation of 1567 mycobacterial isolates included resistance typing, spoligotyping, IS*6110* fingerprinting and typing of the *pks1/15* deletion as described previously [43]–[46]. Mycobacterial strains were for further stratification grouped according to the major phylogenetic lineages [38].

The stepwise procedure of typing of mycobacteria included an initial cluster analysis of IS*6110* fingerprinting data and lineage identification according to specific spoligotype signatures. Assignment of lineages was based on the MIRU-VNTRplus webpage (www.miru-vntrplus.org) and a reference strain collection using the Bionumerics 5.1 software (Applied Maths, Sint-Martens-Latem, Belgium). Classification was confirmed by random selection of 20 strains of each group and testing for the presence of lineage-specific deletions as follows: *pks1/15* for *M. tuberculosis* Euro-American, region of difference (RD) 239 for *M. tuberculosis* East African Indian (EAI), RD9 and RD711 for *M. africanum* West African 1, RD9 and RD702 for *M. africanum* West African 2, and RD9 and RD4 for *M. bovis*. All *M. tuberculosis* strains with ambiguous lineage identification were confirmed as belonging to the Euro-American lineage by identifying the presence of the *pks1/15* 7 bp deletion. Deletion typing was performed using protocols available at the MIRU-VNTRplus webpage.

### MBL Binding Assay

For binding experiments, *M. tuberculosis* aliquots were thawed, spun at 2260× g for 10 min and the pellet was resuspended in TBS buffer (150 mM NaCl, 20 mM TRIS, pH 7.4). To ensure proper dispersion of mycobacteria, 1 ml of the suspension was drawn through a nonpyrogenic needle and aliquots of 1×10^7^ mycobacteria were processed for MBL binding. After centrifugation the pellet was resuspended in 50 µl TBS buffer supplemented with 5 mM Ca^2+^ (TBS/Ca^2+^) and 5 µg/ml recombinant human MBL (R&D Systems, Wiesbaden, Germany) followed by an incubation at 37°C for 30 min. Cells were washed by adding 100 µl TBS/Ca^2+^ buffer and centrifuged before resuspension in 50 µl TBS/Ca^2+^ containing 2.5 µg/ml biotinylated anti-human MBL antibody (R&D Systems, Wiesbaden, Germany) and incubation at 37°C for 30 min. After washing by addition of TBS/Ca^2+^ and centrifugation mycobacteria were resuspended in 50 µl TBS/Ca^2+^ supplemented with Cy5-conjugated streptavidin (Jackson Immunoresearch Europe Ltd., Newmarket, Suffolk, UK) and incubated at 37°C for 30 min. Cells were washed and prepared for flow cytometry by resuspending in 300 µl of TBS/Ca^2+^ buffer and fluorescence intensity was measured on a FACSCalibur™ cytometer at medium flow rates (BD Biosciences, Heidelberg, Germany). The specificity of MBL binding to the clinical isolates was investigated by using the inhibitor EDTA. 15 mM EDTA was added to the TBS/Ca^2+^ buffer 10 min prior to the incubation with MBL. Negative controls comprising mycobacteria processed in the same way but in the absence of MBL were included for every clinical isolate and binding condition.

The data were analyzed using CellQuest Pro software (BD Biosciences, Heidelberg, Germany). In order to discriminate the mycobacterial population from nonbacterial particles a gate was defined in the forward (size) and side scatter (granularity) which was confirmed by mycobacteria labelled with an anti-*Mycobacterium tuberculosis* antibody (abcam, Cambridge, UK). Data were expressed as percentage of cells positive for MBL binding. Non-specific binding was excluded by subtracting the mean percentage of positive cells in the negative controls (w/o MBL) from the respective percentage of positive cells in MBL-treated mycobacteria. Data are representative of four independent experiments which were each performed as triplicate measurements.

### Databases, Statistics

Demographic data, self-reported signs and symptoms documented on structured questionnaires as well as laboratory results were double-entered into a Fourth Dimension database (San Jose, CA, USA). Bacteriological data were provided as Excel datasheets. Data were locked before using them in a pseudonymized form for statistical analyses. Power calculations were performed with the public CATS software (http://www.sph.umich.edu/csg/abecasis/CaTS/).

Multivariate logistic regression analyses were calculated for different models to determine odds ratios (OR) for allele and genotype distributions (STATA 10.0 software; Stata Corporation, College Station, TX, USA). As age, sex and ethnicity were significant confounders, they were appropriately adjusted for. Analyses of allele distributions and Hardy-Weinberg equilibria (HWE) were calculated with a public STATA module (www-gene.cimr.cam.ac.uk/clayton/software/stata/genassoc; David Clayton, Cambridge, UK).

Haplotype frequencies were estimated and compared using the haplo.score module of the haplo.stats package (http://cran.r-project.org/web/packages/haplo.stats/index.html). Max-Stats *P* values obtained testing an overall association between haplotypes and the phenotype corrected for multiple testing by 50,000 permutations.

Differences in the MBL binding properties were calculated using the non-parametric Kruskal-Wallis test.
